# Impact of *Mycobacterium tuberculosis* H37Rv Infection on Extracellular Vesicle Cargo in Macrophages: Implications for Host–Pathogen Interaction

**DOI:** 10.3390/microorganisms12122405

**Published:** 2024-11-23

**Authors:** Manuel G. Salgado-Cantú, Luis Horacio Gutiérrez-González, Silvia Guzmán-Beltrán, María Teresa Herrera, Carmen Sarabia, Yolanda González

**Affiliations:** 1 Department of Microbiology, Instituto Nacional de Enfermedades Respiratorias Ismael Cosío Villegas, Mexico City 14080, Mexico; manuel.salgado@iner.gob.mx (M.G.S.-C.); guzman.silvia@gmail.com (S.G.-B.); teresa_herrera@iner.gob.mx (M.T.H.); mcarmensarabia16@outlook.com (C.S.); 2 Laboratory of Transcriptomics and Molecular Immunology, Instituto Nacional de Enfermedades Respiratorias Ismael Cosío Villegas, Mexico City 14080, Mexico; lhgut@iner.gob.mx

**Keywords:** *M. tuberculosis*, extracellular vesicles, tetraspanin, EV cargo, biomarkers, immune evasion

## Abstract

Tuberculosis (TB) is one of the most common respiratory infections worldwide, and it is caused by *Mycobacterium tuberculosis* (*Mtb*). *Mtb* employs immune evasion mechanisms that allow the disease to become chronic. Despite extensive research, the host–pathogen interaction remains incompletely understood. Extracellular vesicles (EVs) are small membrane particles that play a regulatory role in infectious diseases. Host-derived EVs have been identified as carriers of proteins, messenger RNA, and lipids from both the host cells and the pathogens. In this study, we assessed the cargo of EVs in human macrophages infected with the virulent strain H37Rv of *Mtb* at 1 and 24 h post-infection (hpi). The results showed that 1 hpi, infected macrophages secreted EVs containing *Mtb* proteins (15 to 37 kDa) and Ag85 kDa, as well as RNA transcripts (*ESAT-6*, *5KST*, *Ag85*, *IS6110*, *30 kDa*, *19 kDa*, and *MPT64*). However, these decreased at 24 hpi. The infection of macrophages with *Mtb* was observed to result in the release of EVs containing Ag85 protein and RNA transcripts of *Mtb*; this process appeared to diminish after 24 hpi, suggesting the existence of an evasion mechanism. Both Ag85 and the RNA transcripts could be potential biomarkers for the diagnosis of TB patients.

## 1. Introduction

Although tuberculosis (TB) is a preventable and generally curable disease, an estimated 10.8 million people worldwide were infected with TB in 2023, and 1.25 million people died from the disease. TB is the leading cause of death due to a single infectious agent and the second leading cause of death due to an infectious agent after COVID-19. In 2023, TB probably returned to being the world’s leading cause of death from a single infectious agent, following 3 years in which it was replaced by coronavirus disease. The causative agent, *Mycobacterium tuberculosis* (*Mtb*), is a pathogen with multiple mechanisms to evade the immune system and cause active disease in susceptible individuals but can remain latent in non-susceptible individuals [1].

The H37Rv strain of *Mtb*, which was isolated in 1905, has served as the neotype for the species and has played an important role in understanding the pathogenicity and drug resistance mechanisms of mycobacteria. Despite extensive research, the evasion mechanisms of *Mtb* remain incompletely understood. The possible involvement of extracellular vesicles (EVs) has been investigated under pathological conditions. EVs are small membrane-derived particles (50–100 nm) that are critical mediators of intercellular communication and play an important role in both physiological and pathological conditions. These EVs are characterized by their heterogeneous lipid bilayer membrane containing various biological cargoes, such as cytosol, proteins, lipids, and nucleic acids [2,3], and are present in many body fluids [3]. EVs are resistant to enzymatic degradation during transit. This occurs through the formation of intraluminal vesicles and late endosomes. Macrophage-derivated EVs have the ability to encapsulate a variety of biological molecules, including proteins, messenger RNAs (mRNAs), microRNAs (miRNAs), and DNA, as well as lipids derived from both the parent cell and the pathogen. EVs are then released from cells into the extracellular space, where they modulate the biological function of recipient cells [4].

Host EVs have been identified as carriers of *Mtb* lipids and proteins, further expanding the range of molecules that can be transported between *Mtb* and host cells [5,6]. In latent and active TB patients, it has been reported that *Mtb* resides intracellularly, and its secreted mycobacterial and host RNAs are packaged into host EVs and released into the bloodstream [7,8,9]. In in vitro experiments with *Mtb*-infected RAW264.7 murine macrophages, which is a distinct repertoire of miRNAs, mRNAs were identified; pathogen-derived mycobacterial RNA, *Rv0740*, *Rv0288*, *Rv1344*, *Rv0968*, *Rv1942c*, *Rv0664*, *Rv0190*, *Rv1757c*, and *Rv1369c* proteins were also vesicle-encapsulated and transferred to recipient cells, where they promoted both innate and acquired immune responses [6]. Most studies of EVs and *Mtb* have involved immortalized human monocyte and macrophage or murine cell lines and *Mtb* strains, such as *M. bovis*, *M. avium*, or non-virulent mycobacteria [10,11,12,13]. However, the precise role of EVs in the regulation of the immune system regulation and the persistence of *Mtb* in the host remains poorly understood.

Conversely, it has been documented that macrophage-derived EVs (Mφ-EVs) deliver abundant proteins, lipids, RNA, and DNA that may vary with different macrophage phenotypes or microenvironments, indicating different processes or pathological events [4]. This poses a challenge in elucidating the immunological mechanisms involved when utilizing different experimental models. Here, we used an in vitro model with macrophages and a virulent strain of live *Mtb* (H37Rv). We evaluated some abundant molecules packaged in EVs secreted by the infected macrophages at 1 or 24 h post-infection (hpi) to identify the proteins and RNA of *Mtb* carried in these EVs. The results show that infected macrophages secrete EVs containing *Mtb* proteins and RNA after one hour of infection. However, at 24 hpi, while the number of vesicles remains constant, the composition of the proteins and RNA within them is reduced.

## 2. Materials and Methods

### 2.1. Ethics Statement

The protocol was reviewed and approved by the Institutional Ethics and Research Committee (protocol number: C57-17). All participants were recruited at the National Institute of Respiratory Diseases (INER), Mexico City, and provided written informed consent to participate in the protocol.

### 2.2. Biological Samples

#### 2.2.1. Study Population

Six healthy volunteers (three men and three women) participated in this study. They were vaccinated with BCG at birth and had a positive tuberculin skin test (TST). Five of them were from Mexico City, and one was from Puebla. Mexico is considered a moderate-incidence country for TB.

#### 2.2.2. Peripheral Blood Mononuclear Cell Isolation

Heparinized whole blood (120 mL) was collected from six healthy volunteers, and human peripheral blood mononuclear cells (PBMCs) were gradient purified. Briefly, blood was diluted 1:2 with RPMI 1640 (Lonza, Walkersville, MD, USA), lymphoprep solution (Axis Shield Diagnostics, Oslo, Norway) was added, and then the mixture was centrifuged at 1200 rpm for 45 min at room temperature to obtain PBMCs.

#### 2.2.3. Monocyte Isolation, Culture, and Differentiation

Monocytes were isolated from PBMCs by positive selection using the MACS separation system (Miltenyi Biotec, Auburn, CA, USA) according to the manufacturer’s recommendations. Briefly, PBMCs were mixed with ant-CD14 monoclonal antibody conjugated to magnetic beads (Miltenyi Biotec) and incubated at 4 °C for 15 min. Monocytes were then washed with wash solution (2 mM EDTA, 0.5% bovine serum albumin in PBS 0.01 M, pH = 7.2) and resuspended in wash solution after centrifugation. Monocyte suspension was added to the column under a magnetic field and washed three times. Finally, the column was removed from the magnetic field, and CD14positive cells (monocytes) were eluted. The pure monocyte population was centrifuged and resuspended in an RPMI medium containing 10% heat-inactivated human serum (Valley Biomedical, Winchester, VA, USA). The viability was assessed using trypan blue exclusion (>95%). In addition, the purity of the monocyte population was determined through staining with anti-human CD14-PeTxRed (Invitrogen-Thermo Fisher Scientific, Carlsbad, CA, USA), acquired in a BD FACS Aria flow cytometer, and analyzed using FlowJo software (FlowJo, v10.4.2, Ashland, OR, USA). The number of CD14-positive cells was >90%. Monocytes were resuspended in RPMI medium with 10% heat-inactivated human serum at 1 × 10^6^ MN/mL. A total of 1 mL of the cell suspension was added to 24-well culture plates and incubated at 37 °C in 5% CO_2_ for 7 days to obtain human monocyte-derived macrophages (MDM, also referred to as macrophages).

#### 2.2.4. Human Acute Monocytic Leukemia Cells (THP-1)

Human acute monocytic leukemia cells, THP-1, were obtained from the American Type Culture Collection (ATCC, Manassas, VA, USA). THP-1 cells were grown in RPMI 1640 supplemented with 10% fetal bovine serum (Lonza), 0.05 µM *β*-mercaptoethanol (Bio-Rad, Hercules, CA, USA), and 4 mM L-glutamine (Thermo Fisher Scientific).

### 2.3. Strains and Culture Conditions

The virulent *Mtb* H37Rv strain from the American Type Culture Collection (ATCC 25618, Rockville, MD, USA) was cultivated in Middlebrook 7H9 broth medium (Becton Dickinson, NJ, USA) and stored at −70 °C. Prior to macrophage infection, the bacteria were thawed and disaggregated to obtain a single-cell suspension. The bacteria were centrifuged at 9000× *g* for 10 min, after which the supernatant was discarded and 1 mL RPMI-1640 with glutamine and 5% of exosome-depleted fetal bovine serum (FBS) (SBI, Palo Alto, CA, USA) were added. Then, 3 mm sterile glass beads were added and vortexed for 5 min. The bacterial suspension was then centrifuged at 900× *g* for two minutes. The suspension was then transferred to a new tube for infection assays.

### 2.4. Human Monocyte-Derived Macrophage (MDM/Macrophage) Infection

The medium of the MDM culture was replaced with fresh medium containing 5% of exosome-depleted FBS (SBI, USA) and infected with *Mtb* at a multiplicity of infection (MOI) of 10; uninfected MDM were included as negative controls. Plates were incubated at 37 °C and 5% CO_2_. After 1 hpi or 24 hpi, culture supernatants from infected or uninfected MDM were collected and filtered with sterile 0.22 μm syringe filters (Corning, NY, USA) and stored at 20 °C until EVs isolation.

### 2.5. Isolation of Extracellular Vesicles (EVs)

EVs were isolated from filtered supernatants of uninfected or *Mtb*-infected MDM. Briefly, supernatants were thawed and centrifuged at 2000× *g* for 30 min at 4 °C, and the supernatant was then transferred to a new tube. EVs were then purified from cell supernatants using Total Exosome Isolation Reagent (Invitrogen, Carlsbad, CA, USA) according to the manufacturer’s instructions.

### 2.6. Quantitation of EV Proteins

Individual or pooled EV samples were treated with RIPA lysis solution (Thermo Fisher Scientific) supplemented with EDTA (5 mM) and halt protease inhibitor cocktail (Thermo Fisher Scientific); the mixture was then shaken at 4 °C for 15 min and sonicated for additional 15 s. The samples were centrifuged at 14,000× *g* for 15 min at 4 °C, and the supernatant was collected. The extracted proteins were diluted 1:10 with double-distilled water, and samples of bovine serum albumin standard (Bio-Rad) and the Quick Start Bradford Protein Assay (Bio-Rad) were added to a 96-well flat-bottom plate (Thermo Fisher Scientific). The plate was shaken at 21 °C. The absorbance was measured at 595 nm using an Epoch™ microplate reader (Biotek, Santa Clara, CA, USA).

### 2.7. Expression of CD9, CD63, CD81, and Syntenin in EVs Using Flow Cytometry

Pooled EVs from uninfected MDM (EV-MDM) or infected MDM (EV-MDM-*Mtb*) were incubated with antibodies anti-CD9-PE, anti-CD63-PerCP-Cy5.5, anti-CD81-APC (Biolegend, San Diego, CA, USA), or anti-syntenin-APC (Assaypro, St Charles, MO, USA) for 20 min at room temperature. EVs were then isolated using the ExoQuick kit according to the manufacturer’s instructions and resuspended in filtered PBS containing 1% paraformaldehyde. A total of 200,000 small-particle or diluent events were acquired using a BD FACSymphony A1 (Cell Analyzer, San Jose, CA, USA) and a BD^®^ Small Particle Detector with a High Sensitivity Side Scatter Channel Detector (SP SSC-H) that detects light scattering from small particles, such as EVs. A mixture of Megamix-Plus SSC, fluorescent beads 0.1 to 1 μm (Thermo Fisher Scientific), and SSCs, as a size-dependent parameter (BioCyteX, Marseille, FR), were used for gating. The purity of EVs was analyzed using the FlowJo software.

### 2.8. Internalization of EVs by THP1 Cells

Pooled EV-MDM-*Mtb* were stained with 5(6)-Carboxyfluorescein diacetate N-succinimidyl ester (CFSE) (Sigma Aldrich, Darmstadt, Germany) according to the manufacturer’s instructions. Briefly, CFSE was added to the EVs to a final concentration of 100 μM and incubated for 10 min at 37 °C. The reaction was stopped by adding 3 volumes of cold PBS and incubating the mixture for 5 min on ice; EVs were then precipitated by adding ExoQuickTM reagent (SBI) at 4 °C for 30 min and then centrifuging the mixture at 1500× *g* for 30 min at 4 °C. The supernatant was removed, and the pellet was resuspended in 100 µL of RPMI with 200 mM of L-glutamine and 5% of SBF exosome depleted (Gibco-Thermo Fisher Scientific); 1 × 10^6^ THP1 cells were incubated with 50 µL of the pool of EV-MDM-Mtb-CFSE for one (1 hpi) or twenty-four hours (24 hpi) at 37 °C in 5% CO_2_. The cells were subsequently harvested and centrifuged at 1500 rpm for 5 min at 4 °C. The supernatant was collected and stored at −20 °C, the cells were resuspended in 1x Perm/Wash™ Buffer (BD), anti-human CD9-PE, CD63-PerCpCy5.5, and CD81-APC (Biolegend) were added, and the mixture was incubated for 30 min at room temperature protected from light. Then, 1 mL of washing solution (5% FBS in PBS) was added, the mixture was centrifuged at 1500× *g* for 5 min at 4 °C, and the cells were resuspended in 400 µL of 1% paraformaldehyde in PBS. Cells were acquired on a six-channel ImageStreamX MKII system (Amnis, Fremont, CA, USA) using INSPIRE ver.2 software with brightfield illumination and 488 nm and 642 nm lasers. For compensation, a total of 12,000 events were acquired with the brightfield illuminator and SSC turned off. Cells were selected from the histogram of “Gradient RMS of Channel 1 (brightfield)”, and a plot of “Area of Channel 1” vs. “Aspect Ratio Intensity of Channel 1 (brightfield)” was created from the region in focus. The EVs were selected as fluorescent green spots with a plot of “Intensity of channel 2 (CFSE)” vs. “Max Pixel of channel 2”. From the region of cells with EVs, the EVs inside the cell were selected using the internalization score. From the cells with EVs inside, the cells positive for CD9 (PE) or CD81 (APC) tags were selected using the “Max Pixel of Channel 3 (PE)” vs. “Max Pixel of Channel (APC)” graph.

### 2.9. Identification of Mtb Protein Cargo in EVs Using Western Blotting

A total of 10 μg of either EV-MDM or EV-MDM-*Mtb* protein was combined with Laemmli buffer and β-mercaptoethanol. The proteins were then separated using 4–15% SDS-PAGE and transferred to PVDF membranes. The membranes were probed with polyclonal anti-*Mtb* (donated by Dr. Iris Estrada-García), anti-CD63, or anti-Ag85B (Abcam, Cambridge, UK) antibodies, followed by secondary antibody IgG-HRP (Thermo Fisher Scientific). Immune complexes were identified using the chemiluminescent substrate Clarity (Bio-Rad) and the ChemiDocMP imaging system (Bio-Rad). Total proteins were stained using a silver staining kit (Sigma Aldrich) after SDS-PAGE.

### 2.10. Identification of Mtb RNA Cargo in EVs Using PCR Amplification

According to the manufacturer’s instructions, the total exosomal RNA and protein isolation kit (Invitrogen-Thermo Fisher Scientific) was used to extract RNA from EV-MDM or EV-MDM-*Mtb*. Next, using the RNA as a template, cDNA was synthesized and prepared using the TaqMan MicroRNA Reverse Transcription Kit (Applied Biosystems-Thermo Fisher Scientific) with hexamers (Invitrogen, Thermo Fisher Scientific). A total of 70 ng of cDNA and forward and reverse specific primers (Table 1) for seven mycobacterial gene fragments (*ESAT−6*, *5KST*, *Ag85*, *IS6110*, *30 kDa*, *19 kDa*, and *MPT64*) and rRNA*16S* as the endogenous gene were amplified in a Thermal Cycle (Applied Biosystems-Verity Thermo Fisher Scientific). The amplified fragments were then separated on an agarose gel, stained with Gel Red (Biotum, Fremont, CA, USA), and photographed under UV light using a Chemi-DocMP imaging system (Bio-Rad).

### 2.11. Statistical Analyses

Numbers and percentages are used to represent categorical data without statistical tests. Comparisons between groups were performed by the Kruskal–Wallis test using GraphPad Prism software 10.4.0 version (Boston, MA, USA); *p* ≤ 0.05 was considered significant. The number of positive genes per individual in each group was compared using the Wilcoxon rank sum test (*p* = 0.003) using R-project software version 4.1.2 (2021) [17]. More details can be found in the figure legends.

## 3. Results

### 3.1. Characterization of EVs

**Figure 1:**
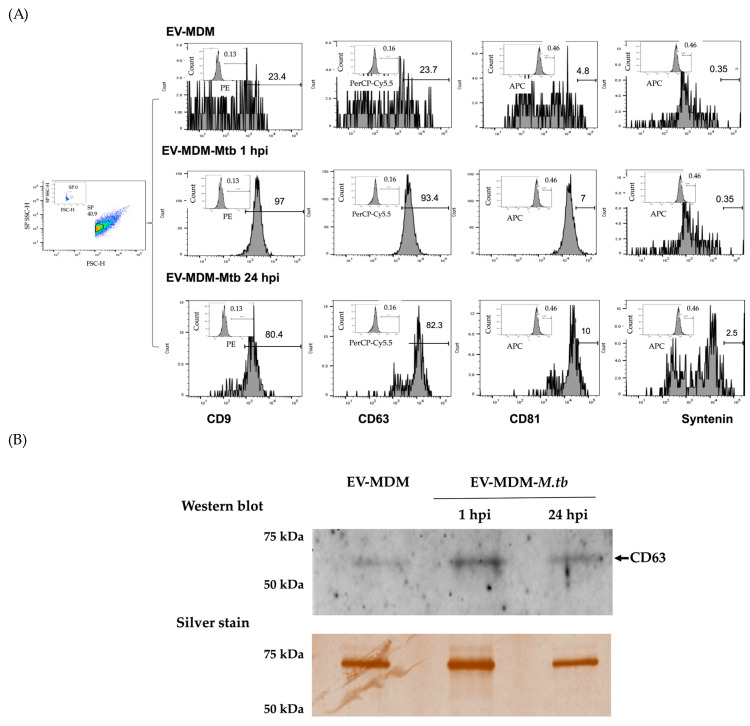

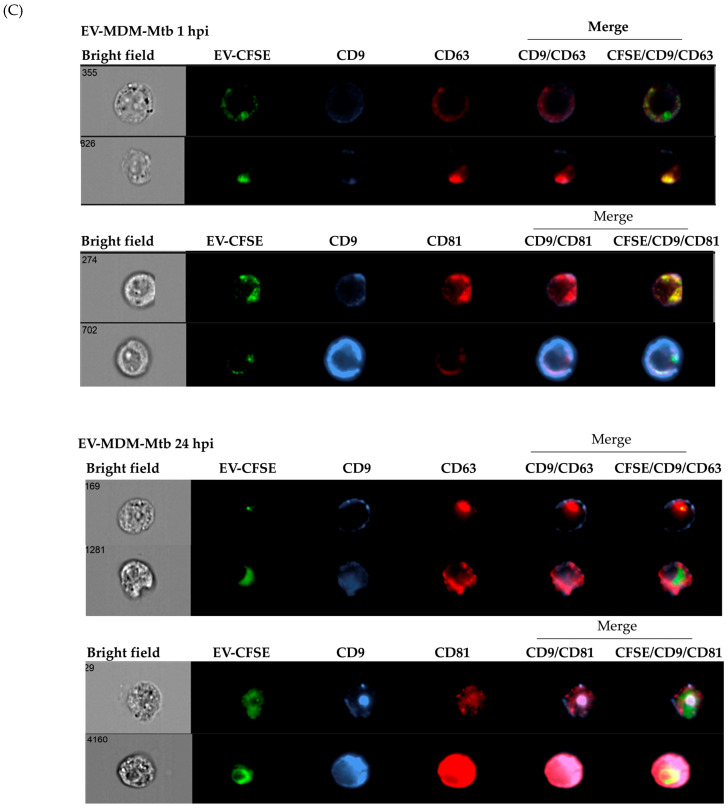
Characterization of pooled EVs from infected MDM. The surface markers CD9, CD63, CD81, and syntenin were detected using flow cytometry (**A**). EV proteins were separated using SDS-PAGE, and CD63 was detected using Western blotting; the lower membrane shows silver-stained total protein as a loading control (**B**). Characterization of EVs inside THP1 cells. EVs-CFSE from *Mtb*-infected MDM (1 or 24 hpi) were added to THP-1 cells, and intracellular vesicles were stained with antibodies against CD9-PE, CD63-PerCP-Cy5.5, and CD81-APC; the expression was detected using an Amnis^®^ imaging flow cytometer. This figure shows a representative image of CD9, CD63, and CD81 expression in EVs (**C**).

We assessed tetraspanin expression on the surface of EVs using flow cytometry and Western blotting. Specific proteins of EVs have been described previously, including CD63, CD9, CD81, and syntenin-1 [18]. Pooled EVs from uninfected MDM expressed CD63, CD9, CD81, and syntenin at rates of 23.7%, 23.4%, 4.8%, and 0.35%, respectively. EVs from MDM infected with *Mtb* after 1 hpi showed high expressions of CD9 (97%) and CD63 (93%) and low expressions of CD81 (7%) and syntenin (0.35%), while EVs from MDM, after 24 hpi, expressed CD9 (80%) and CD63 (82%) and exhibited low expressions of CD81 (10%) and syntenin (2.5%) (Figure 1A). In addition, the expression of CD63 was examined using Western blotting of pooled EVs derived from both uninfected and infected MDM. The CD63 protein was observed in all samples using Western blotting, and the total protein was measured for each sample (Figure 1B). Additionally, pooled EV-MDM-*Mtb* (1 and 24 hpi) were characterized by the expression of CD9, CD63, and CD81 inside THP1 cells (Figure 1C).

### 3.2. Detection of Mycobacterial Proteins in EVs

**Figure 2:**
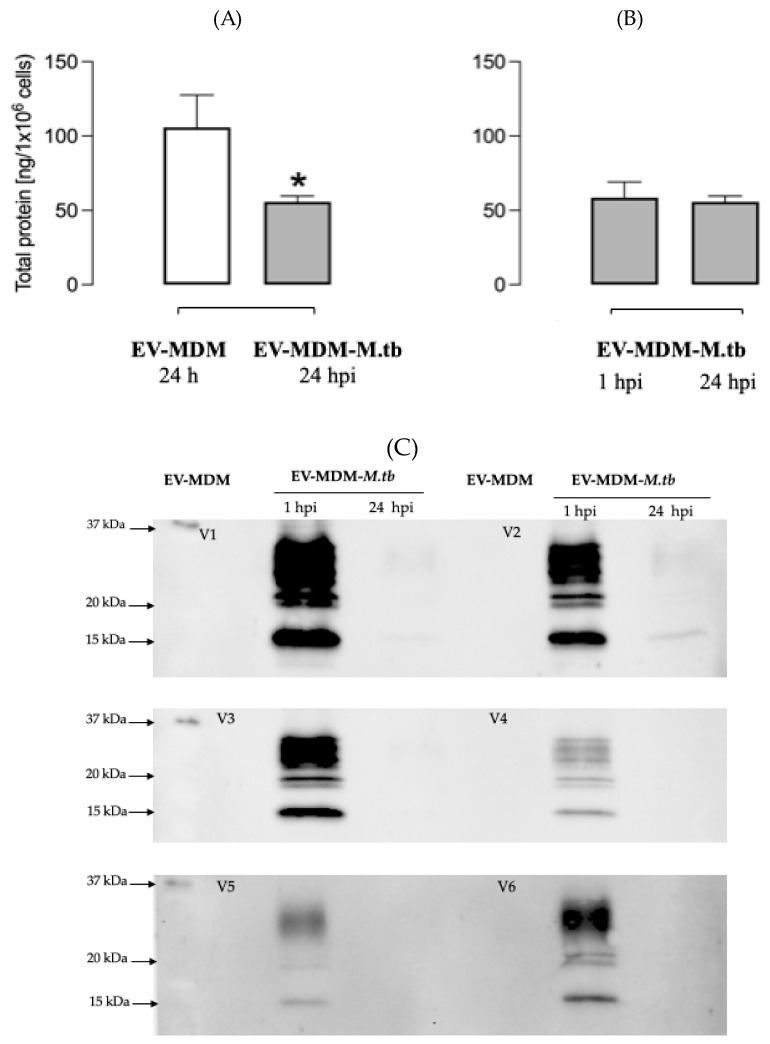
Mycobacterial proteins in EVs from MDM. EVs from six volunteers were isolated from supernatants of MDM at 24 hpi or MDM infected with *Mtb* at 1 or 24 hpi. EVs were lysed and proteins were quantified by Bradford (**A**,**B**), comparisons between groups were performed by the Kruskal–Wallis test, * *p* < 0.05. Proteins were separated using SDS-PAGE and transferred to a PVDF membrane. The membrane was incubated with rabbit polyclonal antibodies against *Mtb* (**C**).

**Figure 3:**
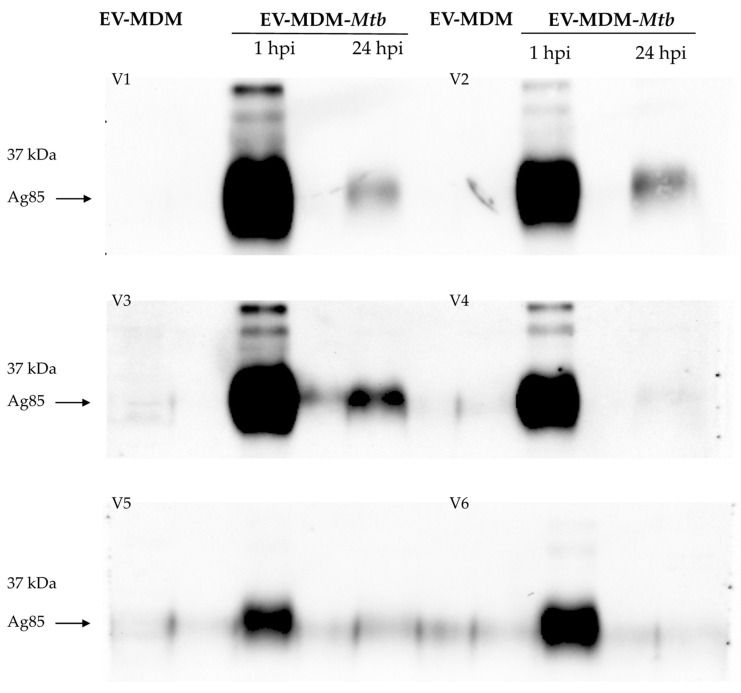
Detection of the mycobacterial antigen Ag85 in EVs. EVs from six subjects were isolated from supernatants of MDM- or MDM-*Mtb*-infected cells (1 or 24 hpi). EVs were lysed and proteins were separated using SDS-PAGE and transferred to a PVDF membrane. The membrane was incubated with an antibody against Ag85 from *Mtb*.

We found that *Mtb* infection greatly reduces the total protein concentration in EVs at both 1 and 24 hpi (Figure 2A); however, we observed distinct mycobacterial proteins in EVs from uninfected or at 1 or 24 hpi cells. Rabbit serum polyclonal antibodies identified mycobacterial proteins, with molecular weights ranging from 15 to 37 kDa in EV-MDM-*Mtb*. At 1 hpi, these proteins were highly expressed, but their expression decreased by 24 hpi. EV-MDM-*Mtb* from volunteers V1, V2, V3, and V6 contained the most proteins (Figure 2B). We then examined CFP-10, ESAT-6, and Ag85 (specific antigens for *Mtb*) in EVs. Ag85 antigen was observed in infected MDM at 1 hpi and was significantly reduced at 24 hpi (Figure 3). CFP-10 and ESAT-6 proteins were not detected in EVs from any subject.

### 3.3. Detection of Mycobacterial RNAs in EVs

Mycobacterial hypothetical protein was present in exosomes from infected RAW264.7 murine macrophages [6]. In addition, we detected RNA from *30 kDa* and *5KST* of *Mtb* in the serum-EVs of TB-MDR patients [8]. Here we evaluate the RNA of the mycobacterial genes *ESAT-6, 5KST*, *Ag85, IS6110*, *30 kDa*, *19 kDa*, *MPT64*, and rRNA*16S* in EVs from infected MDM.

**Figure 4:**
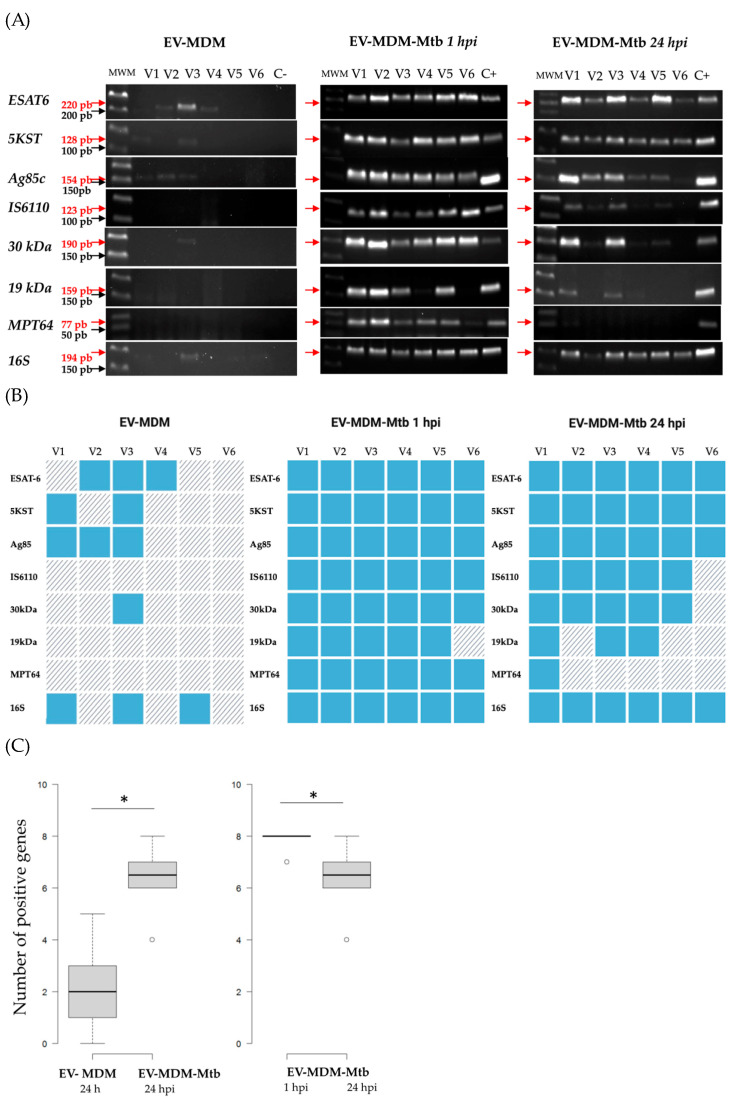
EVs contain RNA sequences of mycobacterial antigens. RNA was extracted from EVs, and cDNA was synthesized, followed by PCR detection of the following mycobacterial genes: *ESAT-6*, *5KST*, *Ag85*, *IS6110*, *30 kDa*, *19 kDa*, *MPT64*, and rRNA*16S*. Fragments were separated using agarose gel electrophoresis, stained, and visualized under UV light. Mycobacterial amplified fragments in EVs from uninfected or *Mtb*-infected MDM (1 or 24 hpi) (A). Heatmap of EVs extracted from six healthy volunteers; blue represents positive amplification, and diagonal gray lines represent negative amplification (B). Box plot of the number of positive genes per individual for each group (Biorender). It shows a significant difference between groups, as follows: * Wilcoxon rank sum test for “EV-MDM” vs. “EV-MDM-*Mtb* 24 hpi” showed *p* = 0.007796 (left graphic), and Wilcoxon rank sum test for “EV-MDM-*Mtb* 1 hpi” vs. “EV-MDM-*Mtb* 24 hpi” showed *p* = 0.0248 (right graphic) (**C**).

**Figure 5:**
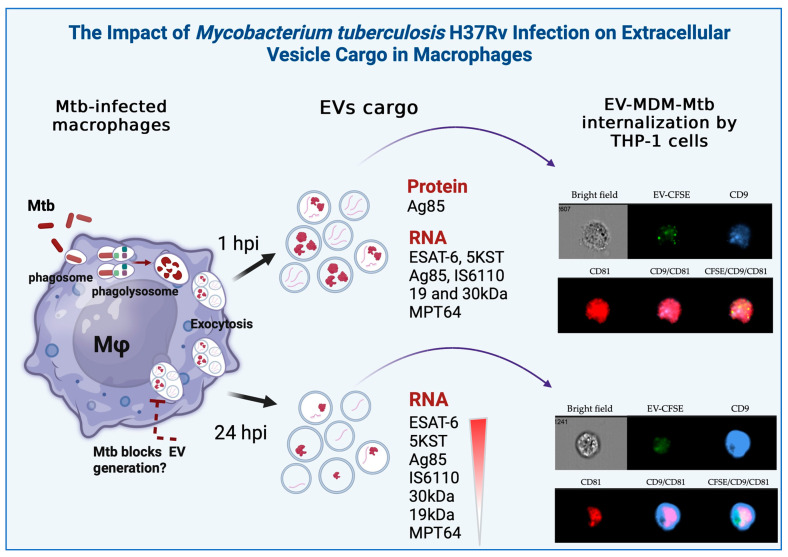
EVs released from *Mtb*-infected cells with the H37Rv *Mtb* strain at 1 hpi carry mycobacterial proteins, as well as RNA transcripts of *ESAT-6, 5KST, Ag85, IS6110, 19 kDa, 30 kDa*, and *MPT64* proteins. These EVs can be inserted into other cells and express tetraspanins, such as CD9, CD63, and CD81. At 24 hpi, *Mtb* arrests phagosome–lysosome fusion and probably prevents the release of EVs carrying *Mtb* antigens and genetic material, as well as tetraspanin expression.

The RNA expression of six healthy volunteers (Figure 4A) and the overall analysis of all volunteers is shown as a heatmap (Figure 4B). *ESAT-6*, *5KST*, *Ag85*, *30 kDa*, and rRNA*16S* were detected in EVs from the uninfected or infected MDM of five healthy volunteers. The mycobacterial *ESAT-6*, *5KST*, *Ag85*, *IS6110*, *30 kDa*, *19 kDa*, *MPT64*, and rRNA*16S* gene fragments were present in EVs at 1 hpi, except V6 for the *19 kDa* fragment. In contrast, at 24 hpi, EVs’ RNA cargo decreased significantly. Specifically, mycobacterial transcripts for *IS6110* and *30 kDa* were not detected in V6; *19 kDa* was not detected in V2, V5, or V6; and *MPT64* was not detected in V2–V6 (Figure 4C). Figure 5 provides a graphical summary of the results of this study.

## 4. Discussion

The role of EVs in regulating or exacerbating non-communicable and infectious diseases has opened new avenues for understanding the progression of diseases and developing new treatment options. In TB, the role of EVs in pathogenesis and biomarker identification has been reported. However, due to the heterogeneity of EV cargo, further studies are required to understand their role in TB.

In this study, EV cargo was examined in human MDM at 1 and 24 hpi with the virulent H37Rv strain of *Mtb.* The EVs obtained from MDM culture supernatants exhibited expression of tetraspanins, with CD9 and CD63 present at approximately 20%. This expression of CD9 and CD63 increased to over 90% and 80% in EVs from MDM after 1 and 24 hpi, whereas CD81 and syntenin increased up to 10 and 2.5% in EVs at 24 hpi. Although the increased EV secretion has been shown in response to a variety of stimuli, including electric stimulation, drug administration, X-ray and UV radiation, sound waves, mitochondrial metabolism, and viruses [19], it is possible that the increased expression of CD9, CD63, CD81, and syntenin may be associated with *Mtb* infection. The role of tetraspanins in TB disease has been studied before. CD81 binds to the *M. abscessus* antioxidant enzyme alkyl hydroperoxidase C (AhpC) to facilitate early macrophage interactions. Given that *Mtb* AhpC shares ∼83 % similarity with *M. abscessus* AhpC, it is possible that CD81 is a receptor for the latter pathogenic mycobacteria and may also facilitate macrophage interactions [20]. The C-terminus of ESAT-6 also binds to the PDZ-domains of syntenin-1, suggesting that the host protein syntenin-1 is a possible cellular receptor for the mycobacterial protein ESAT-6 [21]. Further studies are required to demonstrate whether EVs in conjunction with *Mtb* antigens may be involved in modulating the immune response.

EVs containing *Mtb* proteins or RNA do not necessarily express tetraspanins. It has been reported that *Mtb*-derived EVs are not necessarily packaged in host EVs [22]. This opens an important field for future studies. Here we find that that *Mtb* infection increased the expression of CD9 and CD63 tetraspanins in EVs. Syntenin and tetraspanins are involved in many cellular processes, such as cell adhesion, the regulation of cell motility and/or morphology, fusion, signaling, and other functions, and increasing evidence suggests that they play a central role in the pathogenesis of viral infections [23]. Additionally, it has been reported that during *Mtb* infection, the lysosomal glycoprotein CD63 is recruited to *Mtb* phagosomes [24]. Based on this, it is possible to speculate that *Mtb* could evade immune responses by modulating the expression of syntenin and tetraspanins.

The total protein in EVs from infected MDM was not associated with a decrease in the abundance of *Mtb* proteins, as our results showed that some mycobacterial proteins were present in EVs at 1 hpi, whereas these proteins were significantly reduced at 24 hpi. Previous studies have reported more than 41 mycobacterial proteins in EVs released from *Mtb*-infected J774 macrophages using highly sensitive techniques, such as proteomics [25], but the transport of mycobacterial proteins in EVs at different infection stages remains to be evaluated. In another study using non-virulent *M. bovis* BCG–infected J774 macrophages, it was reported that lipoarabinomannan and the 19 kDa lipoprotein took between 72 and 120 h to appear [26]. In contrast, in virulent *Mtb* strains, we only detected the RNA of these proteins at an early stage of infection, followed by a decrease in 19 kDA lipoprotein RNA. Furthermore, our results showed a decrease in Ag85 kDa after 24 hpi. We investigated the presence of *Mtb* Ag85 kDa in EVs, as Ag85A kDa and Ag85B kDa account for approximately 60% of the total protein secreted by *Mtb* [27]. Previous studies have reported that Ag85B is downregulated during chronic infection, inducing CD4+ T cells to recognize the Ag85B epitope [28]. This suggests that this decrease may be associated with an immune evasion mechanism of *Mtb*. However, this is an issue that requires further investigation.

RNA transcripts have been reported to originate from vesicles released by mycobacteria during infection. Both host and mycobacterial small RNA fragments have been identified in *Mtb*-infected macrophages and patients with TB [6,8]. Mycobacterial transcripts have been identified in exosomes/EVs using the RAW264.7 murine macrophage [6]. Here, we evaluated transcripts previously studied in TB patients and found that *ESAT-6*, *5KST*, *Ag85*, *IS6110*, *30 kDa*, *19 kDa*, and *MPT64* were expressed in EVs. As with *Mtb* proteins, RNA transcripts decreased after 24 hpi, with a decrease in *MPT64* observed in most patients, followed by the *19 kDa* transcript and, in some individuals, the *30 kDa* transcript and *IS6110*. The function of mycobacterial RNAs as pro-inflammatory mediators and in recruiting cells to sites of infection is well-established [6,29]. However, further studies are needed.

This was an unexpected demonstration that RNA transcripts of *ESAT-6*, *5KST*, *Ag85*, and *30 kDa* were present in EVs from uninfected healthy subjects. This finding may indicate the possibility of latent infection or exposure to *Mtb*, as all subjects were from TB-endemic areas and were TST-positive. Previously, it has been reported that *Mtb* occurs intracellularly; its secreted mycobacterial antigens and host RNAs are packaged into host EVs and released into the bloodstream. In addition, *Mtb* proteins have been detected in healthy individuals, possibly with prior infections [30,31]. RNA transcripts of *Mtb* have also been detected in both healthy and latently TB-infected individuals [32]. It is difficult to determine whether the healthy subjects tested were exposed to *Mtb* or latently infected. Further analysis may provide more insight into this issue.

One of the limitations in studying EVs from healthy human subjects or patients is the number of EVs that can be obtained using assays. While the use of cell lines can provide a distinct advantage for their analysis, the response of primary cells and the inherent variability in response can only be elucidated by studying primary cells from healthy subjects.

## 5. Conclusions

*Mtb* infection resulted in the increased expression of the CD9 and CD63 tetraspanins in EVs released by macrophages. EVs carry Ag85-*Mtb* and various RNA sequences from *Mtb* in the early stages of infection; however, the EV cargo appears to diminish by 24 hpi. Given that *Mtb* is known to evade phagosome–lysosome fusion, the reduction in EV cargo may be linked to this evasion mechanism. Both Ag85 and the RNA transcripts could therefore serve as potential biomarkers for diagnosing *Mtb* infection or latency.

## Figures and Tables

**Table 1:** Mycobacterial gene primers.

Target	Primer Sequences	Size (bp)	Reference
*ESAT-6*	F: GTCCATTCATTCCCTCCT R. CTATGCGAACATCCCAGT	220	[14]
*5KST*	F: TTGCTGAACTTGACCTGCCCGTA R: GCGTCTCTGCCTTCCTCCGAT	128	[15]
*Ag85*	F: AAGGTCCAGTTCCAGGGCG R: ATTGGCCGCCCACGGGCATGAT	154	[8]
*IS6110*	F: CCTGCGAGCGTAGGCGTCGG R: CTCGTCCAGCGCCGCTFCGG	123	[16]
*30 kDa*	F: TGTACCAGTCGCTGTAGAAG R: GACATCAAGGTTCAGTTCC	190	[14]
*19 kDa*	F: GAGACCACGACCGCGGCAGG R: AATGCCGGTCGCCGCCCCGCCGAT	159	[8]
*MPT64*	F: GTGAACTGAGCAAGCAGACCG R: GTTCTGATAATTCACCGGGTCC	77	[8]
*rRNA* *16S*	F: GCCGTAAACGGTGGGTACTA R: TGCATGTCAAACCCAGGTAA	194	[8]

## Data Availability

The original contributions presented in the study are included in the article, further inquiries can be directed to the corresponding author.
